# Effects of photobiomodulation on the differentiation, viability, and migration of C2C12 myoblasts exposed to different concentrations of dexamethasone

**DOI:** 10.1038/s41598-026-52078-6

**Published:** 2026-05-13

**Authors:** Alessandra Lima da Silva Martins, Tainá Caroline dos Santos Malavazzi, Rosani Tereza de Siqueira e Silva, Claudio Teruo Kassa, Mohammadhossein Shaker, Aline Souza Silva, Cinthya Cosme Gutierrez Duran, Anna Carolina Ratto Tempestini Horliana, Sandra Kalil Bussadori, Kristianne Porta Santos Fernandes, Raquel Agnelli Mesquita-Ferrari

**Affiliations:** 1 Postgraduate Program in Medicine-Biophotonics, Nove de Julho University (UNINOVE), 235/249 Vergueiro Street, Liberdade, São Paulo, 01504-001, 01525-000 Brazil; 2 Department of Stomatology, Discipline of Oral and Maxillofacial Pathology, School of Dentistry, University of São Paulo (FOUSP), São Paulo, 05508-000 SP Brazil; 3 Present Address: Postgraduate Program in Rehabilitation Sciences, Nove de Julho University (UNINOVE), 235/249 Vergueiro Street, Liberdade, Sao Paulo, 01504-001 Brazil

**Keywords:** atrophy, muscle cells, C2C12, dexamethasone, photobiomodulation, Biological techniques, Biotechnology, Cell biology, Medical research

## Abstract

Glucocorticoid-induced muscle atrophy remains a clinical concern because of its broad impact on skeletal muscle structure, metabolism, and regenerative capacity. This study examined whether photobiomodulation (PBM) could lessen the damaging effects of dexamethasone (DEXA) on C2C12 myoblasts during differentiation. Cells were exposed to 5, 10, or 200 µM DEXA and evaluated after 24, 48, and 72 h. A single PBM session was delivered using a 780-nm laser (70 mW, 1.05 J, 26.25 J/cm^2^). Cell viability and proliferation were assessed by MTT and crystal violet assays; differentiation was analyzed through nuclear fusion and morphological staining; migration was measured using the wound-healing assay; and IL-6 and TNF-α levels were quantified by ELISA. DEXA reduced C2C12 viability, fusion, and migration in a time- and dose-dependent manner, particularly at 10 and 200 µM. PBM alone was not cytotoxic and, when combined with DEXA, partially preserved viability, enhanced proliferation, and supported early differentiation, although without restoring control levels. Migration was also mitigated, but not fully recovered. PBM further moderated the DEXA-induced reduction in IL-6 and TNF-α. Overall, PBM mitigated the effects of glucocorticoid-induced stress, indicating its potential as a complementary approach to help preserve myogenic function.

**Supplementary Information:**

The online version contains supplementary material available at 10.1038/s41598-026-52078-6.

## Introduction

Skeletal muscle plays a fundamental role in posture, locomotion, and energy metabolism^1,2^. Its structural and functional integrity relies on a finely regulated balance between proliferation, differentiation, and regeneration of muscle cells^3^. This process is primarily mediated by satellite cells, which are activated in response to injury or mechanical overload and differentiate into myoblasts capable of forming or repairing muscle fibers^4,5^. Muscle injuries are among the most frequent traumas in physically active individuals and demand effective therapeutic strategies to ensure complete recovery^6^. In addition to trauma, several pathological or pharmacological conditions may lead to loss of muscle mass and function, including aging, chronic diseases, and prolonged drug administration^7^.

Dexamethasone (DEXA) is a potent synthetic glucocorticoid widely prescribed for its anti-inflammatory and immunosuppressive effects in the management of inflammatory and autoimmune disorders, organ transplants, and certain malignancies^8,9^. Despite its therapeutic efficacy, long-term or high-dose use is strongly associated with adverse outcomes, including immunosuppression, insulin resistance, and muscle atrophy^10,11^. Muscle atrophy is characterized by a reduction in fiber size resulting from increased protein degradation, leading to a decline in muscle mass and contractile performance^12,13^. The chemical structure of DEXA provides greater stability and affinity for the receptor than endogenous glucocorticoids, producing prolonged pharmacological effects^14^. By binding to the glucocorticoid receptor, DEXA triggers transactivation and transrepression mechanisms that mediate its metabolic and anti-inflammatory actions, respectively. This duality explains the coexistence of therapeutic and deleterious effects observed in clinical contexts^15^.

In muscle cells, DEXA promotes proteolysis, oxidative stress, and suppression of myogenic regulatory factors, ultimately impairing proliferation, differentiation, and regeneration^9,13,16^. Several studies have investigated its effects in C2C12 myoblasts using phytotherapeutic interventions. For instance, total ginseng protein and ginsenoside Rg3 have been shown to mitigate DEXA-induced cytotoxicity by improving mitochondrial function and reducing atrophy-related markers^17,18^. Similarly, curcuminoids from Curcuma longa decreased the expression of Atrogin-1 and MuRF-1, suggesting inhibition of proteolytic pathways^19^. Interestingly, the impact of DEXA appears to vary with the differentiation stage: while it can enhance fusion and structural protein expression in early myoblasts, it induces atrophy in mature myotubes by upregulating catabolic markers^20^. In dysferlinopathy models, DEXA even partially restored myogenic fusion, indicating that its cellular effects can be context-dependent and modulated by additional interventions^21^. Although DEXA remains a valuable therapeutic agent, its long-term administration requires careful management due to its catabolic impact on skeletal muscle. Understanding the molecular basis of these effects is critical for designing strategies to minimize tissue damage and improve recovery^10,13,14,22^.

Photobiomodulation (PBM) therapy uses low-intensity light to stimulate cellular activity and has demonstrated anti-inflammatory and analgesic benefits in various clinical contexts^23^. PBM primarily targets mitochondrial cytochrome c oxidase, enhancing ATP synthesis, modulating reactive oxygen species (ROS) and nitric oxide (NO), and activating key signaling pathways such as NF-κB, PI3K/Akt, and ERK—mechanisms associated with cytoprotection and tissue repair^24^. In C2C12 muscle cell models, PBM has been shown to increase cell viability, proliferation, and differentiation, even under catabolic conditions^25,26^. Its effects appear to depend on irradiation parameters, supporting the notion that PBM acts as a bio-stimulatory agent capable of preserving muscle structure and function^23,27,28^. Moreover, PBM attenuates oxidative stress, decreases the expression of pro-inflammatory cytokines such as IL-6 and TNF-α, and helps maintain muscle fiber integrity^29^.

Previous studies from our research group have demonstrated that PBM, using either lasers or LEDs, modulates C2C12 cell behavior. In a myoblast transplantation model, PBM improved tissue repair by enhancing early macrophage and vascular responses and reducing myonecrosis, indicating regulation of multiple phases of regeneration^30^. In cocultures of J774 macrophages and C2C12 myoblasts, PBM enhanced anti-inflammatory and regenerative responses^25^. In LPS-stimulated myoblasts, PBM (780 nm) modulated viability in a dose-dependent manner and reduced NO synthesis without altering IL-6 levels, suggesting effects primarily linked to bioenergetic and redox pathways^31^.

In another study, exposure of C2C12 cells to infrared light also induced AMPK and p38 MAPK phosphorylation, increasing the expression of PGC-1α and SIRT1, key regulators of mitochondrial biogenesis and myogenic differentiation^32^.

These findings show the positive effects of PBM as a promising therapeutic approach for stimulating muscle regeneration. However, its potential to counteract glucocorticoid-induced atrophy has not yet been investigated. Therefore, this study aimed to evaluate the effects of PBM on differentiation, viability, migration, and cytokine synthesis in C2C12 muscle cells exposed to different concentrations of DEXA during the induction of differentiation.

## Materials and methods

### Cell culture and induction of differentiation

Myogenic C2C12 cells (ATCC^®^ CRL-1772™), derived from mice, were cultured in Dulbecco’s Modified Eagle Medium (DMEM; Vitrocell, Campinas, SP, Brazil), pH 7.4, supplemented with 10% fetal bovine serum (FBS) to induce proliferation and with 2% horse serum (HS) to induce differentiation. The cultures were incubated at 37 °C in a humidified atmosphere containing 5% CO₂. For subculturing, cells were detached using 0.1% trypsin–EDTA, centrifuged at 1200 rpm for five minutes, counted, and allocated to receive the specific treatments corresponding to the experimental groups.

## Experimental groups

Treatments were applied concomitantly with the onset of differentiation, and the cells were allocated into the following experimental groups: (1) Control (cells cultured in differentiation medium without treatment); (2) PBM (cells subjected to PBM only); (3) DEXA 5 µM; (4) DEXA 10 µM; (5) DEXA 200 µM; (6) DEXA 5 µM + PBM; (7) DEXA 10 µM + PBM; and (8) DEXA 200 µM + PBM. In the DEXA groups, cells were cultured in differentiation medium supplemented with the respective concentration of dexamethasone (5, 10, or 200 µM), whereas the combined groups received both PBM and the corresponding DEXA concentration.

### DEXA treatment

DEXA disodium phosphate (C₂₂H₂₈FNa₂O₈P; 516.41 g/mol) was used in the form of an injectable solution at 4 mg/mL (Decadron^®^, Aché Laboratórios Farmacêuticos S.A., São Paulo, Brazil). Experimental concentrations of 5^11^, 10^20^, and 200 µM^18^ were obtained by diluting the stock solution in DMEM supplemented with 2% horse serum (HS) under aseptic conditions to ensure mixture homogeneity. The desired final volume (µL) and concentration (µM) were calculated using the following formula: C₁V₁ = C₂V₂.

### PBM treatment

C2C12 cells were first collected and centrifuged to obtain a cell pellet in sterile conical Falcon tubes^33^. The pellet was then irradiated using a GaAlAs diode laser (Twin Laser^®^, MM Optics, São Carlos, SP, Brazil) with the parameters described in Table 1^25^. Immediately after irradiation, the cells were resuspended in differentiation medium (DMEM supplemented with 2% horse serum) containing DEXA at the respective experimental concentrations (5, 10, or 200 µM) and plated for the subsequent assays.

**Table 1:** Dosimetric parameters used for PBM treatment.

Parameter	Value
Wavelength (nm)	780
Spectral bandwidth (nm)	10
Operating mode	continuous
Power(mW)	70
Polarization	random
Aperture diameter (cm)	0.23
Irradiance at aperture (W/cm^2^)	1.75
Beam area (cm^2^)	0.04
Time (s)	15
Radiant exposure (J/cm^2^)	26.25
Number of irradiated points	1
Irradiated area (cm^2^)	0.04
Application technique	contact
Number of sessions	1
Total energy (J)	1.05

### Cell viability assay

Cell viability was assessed using the MTT assay (3-[4.5-dimethylthiazol-2-yl]-2.5-diphenyltetrazolium bromide; Sigma). Cells (1 × 10⁴ per well in a 96-well plate) were incubated at 37 °C with 5% CO₂. Treatments were applied at the beginning of the experiment according to the experimental groups, and cell viability was evaluated after 24, 48, and 72 h of incubation. The wells were washed with 1× PBS buffer (NaCl 140 mM; KCl 2.5 mM; Na₂HPO₄ 8 mM; KH₂PO₄ 1.4 mM; pH 7.4), followed by the addition of 50 µL of MTT solution (0.5 mg/mL) to each well. Plates were then incubated for an additional 3 h. Subsequently, the formazan crystals were solubilized by adding 100 µL of isopropanol per well. Absorbance was measured at 620 nm using a microplate reader (Anthos2020™, Anthos Labtec Instruments, Wals, Austria).

### Cell proliferation assay

Cell proliferation was evaluated using the crystal violet assay^25^. Cells (1 × 10⁴ per well in 96-well plates) were incubated at 37 °C with 5% CO₂ for 24, 48, and 72 h. A volume of 40 µL of crystal violet solution (5% w/v in 20% v/v methanol; Synth, São Paulo, Brazil) was added to each well corresponding to the experimental period, followed by incubation at room temperature for 15 min. The wells were then washed with distilled water, and the cells were lysed by adding 100 µL of methanol per well. After homogenization, absorbance was measured at 620 nm using a microplate reader (Anthos2020™).

### Fusion index

The fusion index was calculated using the May-Grunwald and Giemsa staining technique^34,35^. Cells (1 × 10⁴ per well in 96-well plates) were incubated at 37 °C with 5% CO₂ for 24, 48, and 72 h. After each period, cells were fixed with methanol (Synth) for 5 min and subsequently stained with May-Grunwald (Renylab, MG, Brazil) diluted 1:3 in sodium phosphate buffer (1 mM NaH₂PO₄ and 1 mM Na₂HPO₄, pH 5.8) for 5 min, followed by Giemsa (Renylab, MG, Brazil) diluted 1:20 in the same buffer for 10 min. Plates were washed with 1X PBS and air-dried at room temperature. Images were captured using an inverted microscope (Eclipse TE300, Nikon, Tokyo, Japan – 40X). Nuclei were counted using ImageJ (NIH), and the fusion index was calculated as the number of nuclei in binucleated and multinucleated cells divided by the total number of nuclei, multiplied by 100.

### Cell migration assay

The in vitro wound healing assay was used to evaluate cell migration^36^. Cells (1.4 × 10⁵ per well in 6-well plates) were incubated at 37 °C with 5% CO₂. After 24 h, a linear scratch was made in the cell monolayer using a 200 µL pipette tip to create the wound. Treatments were applied immediately after scratch formation according to the experimental groups. Images were captured at 0, 6, 12, and 24 h using an inverted microscope (Eclipse TE300, Nikon, Tokyo, Japan) at 40× magnification, and the wound area was measured with ImageJ^27^. The cell migration rate was expressed as the percentage of wound closure, calculated using the formula^37^:

$$\:Wound\:Closure\:\% \:{\text{ = }}\:\left[ {\frac{{{\mathrm{A}}_{{{\text{t = 0h}}}} {\text{ - }}\:{\mathrm{A}}_{{{\text{t = }}\Delta \:{\mathrm{h}}}} }}{{{\mathrm{A}}_{{{\text{t = 0h}}}} }}} \right] \times \:\:{\mathrm{100}}\%$$

Where A_t=0 h_ corresponds to the initial wound area measured immediately after scratching, and A_tΔh_ represents the remaining area after the incubation period. Thus, higher values indicate greater cell migration and, consequently, a higher percentage of wound closure.

### IL-6 and TNF-α cytokine quantification

After the experimental periods, levels of interleukin-6 (IL-6) and tumor necrosis factor-alpha (TNF-α) in the supernatants were determined using ELISA^38–40^. The following kits were used: BioLegend Mouse IL-6 ELISA MAX™ Standard Sets (431301) and BioLegend Mouse TNF-α ELISA MAX™ Standard Sets (430901; Pacific Heights Blvd., San Diego, CA), following the manufacturer’s instructions. Total protein in the supernatant from all experimental groups was quantified using a NanoDrop 2000 (ThermoScientific, Waltham, MA), and the results were used to normalize the IL-6 and TNF-α ELISA^25^.

### Statistical analysis

Data were analyzed using GraphPad Prism 8.0 (GraphPad Software, San Diego, CA, USA). All experiments were performed in three independent experiments, each conducted with five replicates per experimental group, and comparisons between treatments were assessed using one-way analysis of variance (ANOVA). Data are expressed as mean ± standard error of the mean (SEM). Statistical significance was determined by Tukey’s post hoc test, and *p* < 0.05 was considered statistically significant. In addition, figure legends were revised to consistently report data as mean ± SEM.

## Results

### Cell viability

**Fig. 1:**
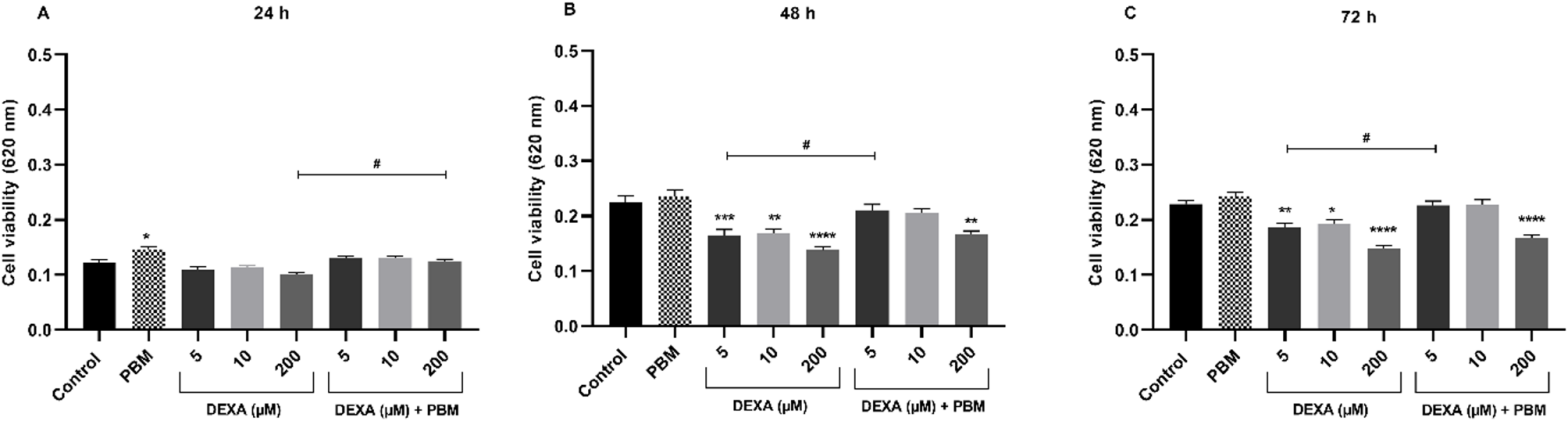
Cell viability of C2C12 myoblasts exposed to DEXA and/or PBM for 24 h (**A**), 48 h (**B**), and 72 h (**C**), assessed by the MTT assay. A time- and concentration-dependent reduction in cell viability was observed in the DEXA-treated groups, whereas PBM partially attenuated these effects. Data are expressed as mean ± SEM. Significant differences compared to the control group are indicated by asterisks (*), and significant differences between groups treated with the same DEXA concentration are indicated by hashtags (#). ANOVA followed by Tukey’s test: *p < 0.05; **p < 0.01; ***p < 0.001; ****p < 0.0001; #*p* < 0.05; ##*p* < 0.01; ###*p* < 0.001; ####*p* < 0.0001.

Cell viability remained comparable to the control during the first 24 h, regardless of DEXA concentration, and the addition of PBM did not alter this initial pattern. After 48 h, DEXA reduced cell viability compared with the control group, with similar effects observed for the 5 and 10 µM concentrations and a more pronounced reduction at 200 µM. Under these conditions, PBM provided partial protection, resulting in higher viability than in the corresponding DEXA-only groups. By 72 h, the decline in viability became more evident, particularly at 200 µM, while PBM continued to partially preserve viability at the lower concentrations. Even so, none of the combinations were able to fully restore the values observed in the control group (Fig. 1).

### Cell proliferation

**Fig. 2:**
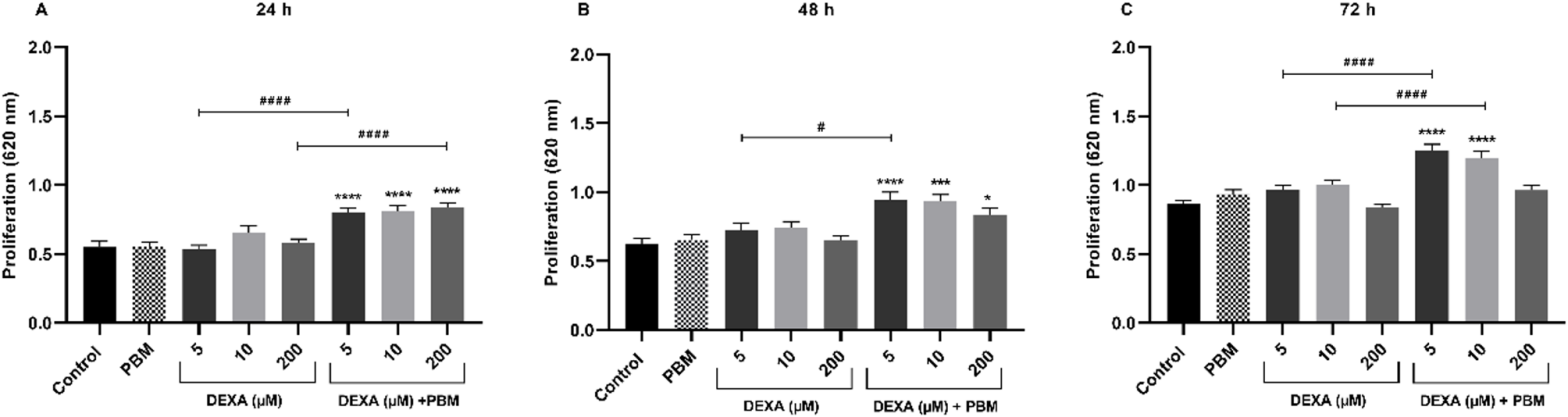
Cell proliferation of C2C12 myoblasts evaluated by the crystal violet assay after 24 h (**A**), 48 h (**B**), and 72 h (**C**) of exposure to DEXA at different concentrations, alone or combined with PBM. Data are expressed as mean ± SEM. Significant differences compared to the control group are indicated by asterisks (*), and differences between groups treated with the same DEXA concentration are indicated by #. Statistical analysis was performed using one-way ANOVA followed by Tukey’s post hoc test: *p < 0.05; **p < 0.01; ***p < 0.001; ****p < 0.0001; #*p* < 0.05; ##*p* < 0.01; ###*p* < 0.001; ####*p* < 0.0001.

Analysis of cell proliferation (Fig. 2) showed that DEXA alone did not significantly affect C2C12 proliferation at any of the evaluated time points. In contrast, its combination with PBM consistently enhanced cell growth. At 24 h, all DEXA + PBM groups exhibited higher proliferation than their corresponding DEXA-only groups. At 48 and 72 h, the association with PBM resulted in significantly increased proliferation compared with the control, with the 5 and 10 µM combinations also surpassing their respective DEXA-only conditions. Taken together, these findings indicate that PBM supports C2C12 proliferation even in the presence of DEXA.

### Fusion index

**Fig. 3:**
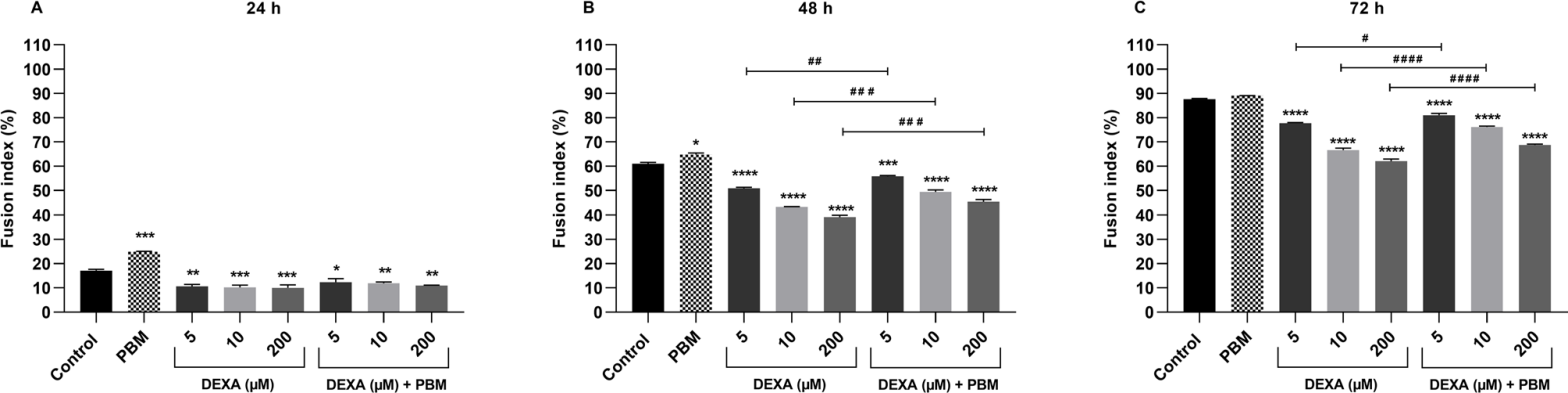
Fusion index (%) of C2C12 myoblasts exposed to DEXA and/or PBM for 24, 48, and 72 h. Data are expressed as mean ± SEM. Significant differences compared to the control group are indicated by asterisks (*), and differences between groups treated with the same DEXA concentration are indicated by hashtags (#). Statistical analysis was performed using one-way ANOVA followed by Tukey’s post hoc test: *p < 0.05; **p < 0.01; ***p < 0.001; ****p < 0.0001; #*p* < 0.05; ##*p* < 0.01; ###*p* < 0.001; ####*p* < 0.0001.

The fusion index of C2C12 cells showed a consistent decline in all groups treated with DEXA across the evaluated time points (24, 48, and 72 h), regardless of dose (Fig. 3). At 24 h, none of the DEXA or DEXA + PBM groups reached values comparable to the control, whereas PBM alone produced a significant increase in fusion. At 48 h, DEXA continued to suppress myotube formation, but its combination with PBM led to a partial recovery of the fusion index when compared with DEXA alone, indicating a mitigating effect. A similar pattern was observed at 72 h, PBM reduced part of the impairment but did not restore control levels. Overall, these findings show that DEXA persistently compromises myoblast fusion, while PBM supports early differentiation events and partially counteracts these deleterious effects.

**Fig. 4:**
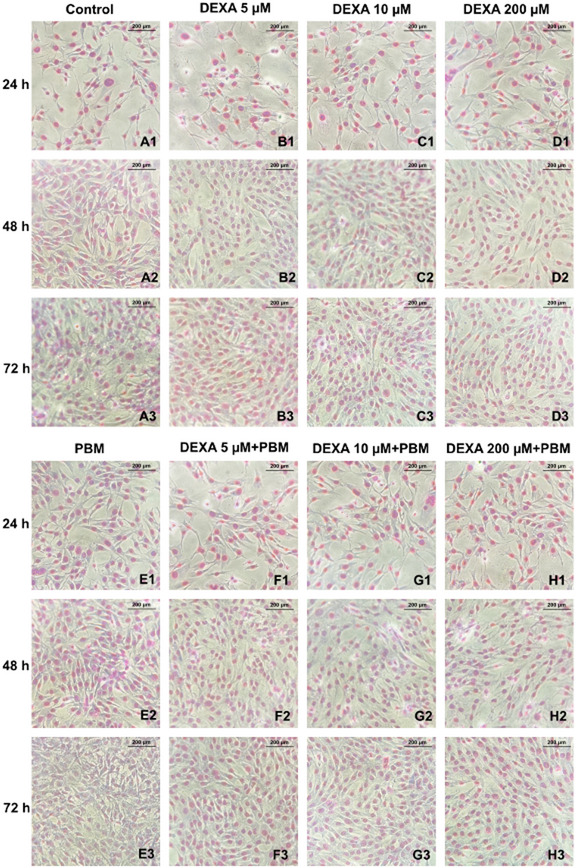
May-Grünwald and Giemsa staining of C2C12 myoblasts exposed to DEXA and/or PBM for 24, 48, and 72 h. The images show cell morphology and nuclear fusion throughout experimental periods. (A1–A3) Control; (B1–B3) DEXA 5 µM; (C1–C3) DEXA 10 µM; (D1–D3) DEXA 200 µM; (E1–E3) PBM; (F1–F3) DEXA 5 µM + PBM; (G1–G3) DEXA 10 µM + PBM; (H1–H3) DEXA 200 µM + PBM. DEXA, especially at 200 µM, reduced myoblast fusion, whereas PBM partially attenuated these effects, promoting a higher number of multinucleated myotubes. Magnification 40×; Scale bar = 200 μm.

Morphological analysis showed that control cells maintained the expected features of myogenic differentiation, exhibiting progressive elongation, increasing alignment, and clear formation of myotube-like structures by 72 h (Fig. 4). In the DEXA-treated groups, structural alterations were evident at all time points, including reduced elongation, poorer alignment, and a higher proportion of rounded cells, with these effects becoming more pronounced at higher concentrations and longer treatment durations. PBM alone preserved a morphology consistent with normal differentiation, while its combination with DEXA partially mitigated the drug-induced alterations, resulting in a greater number of elongated and better-organized cells compared with DEXA-only groups. Nevertheless, at higher DEXA concentrations and after 72 h, signs of impaired morphology remained when compared with the control condition.

### Cell migration

**Fig. 5:**
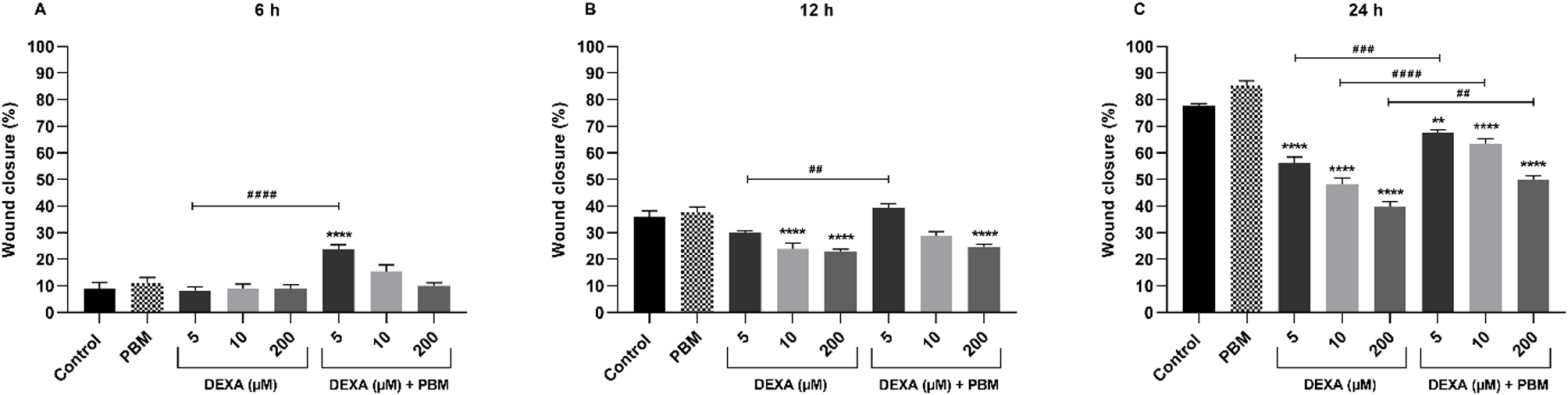
Wound closure (%) in C2C12 cells according to experimental groups at 6 h (**A**), 12 h (**B**), and 24 h (**C**) of exposure to DEXA at different concentrations, either alone or in combination with PBM. Data are presented as mean ± SEM. Significant differences compared to the control group are indicated by asterisks (*), and significant differences between groups treated with the same dexametasone concentration are indicated by hashtags (#). ANOVA followed by Tukey’s test: *p < 0.05; **p < 0.01; ***p < 0.001; **p < 0.0001; #*p* < 0.05; ##*p* < 0.01; ###*p* < 0.001; ####*p* < 0.0001.

**Fig. 6:**
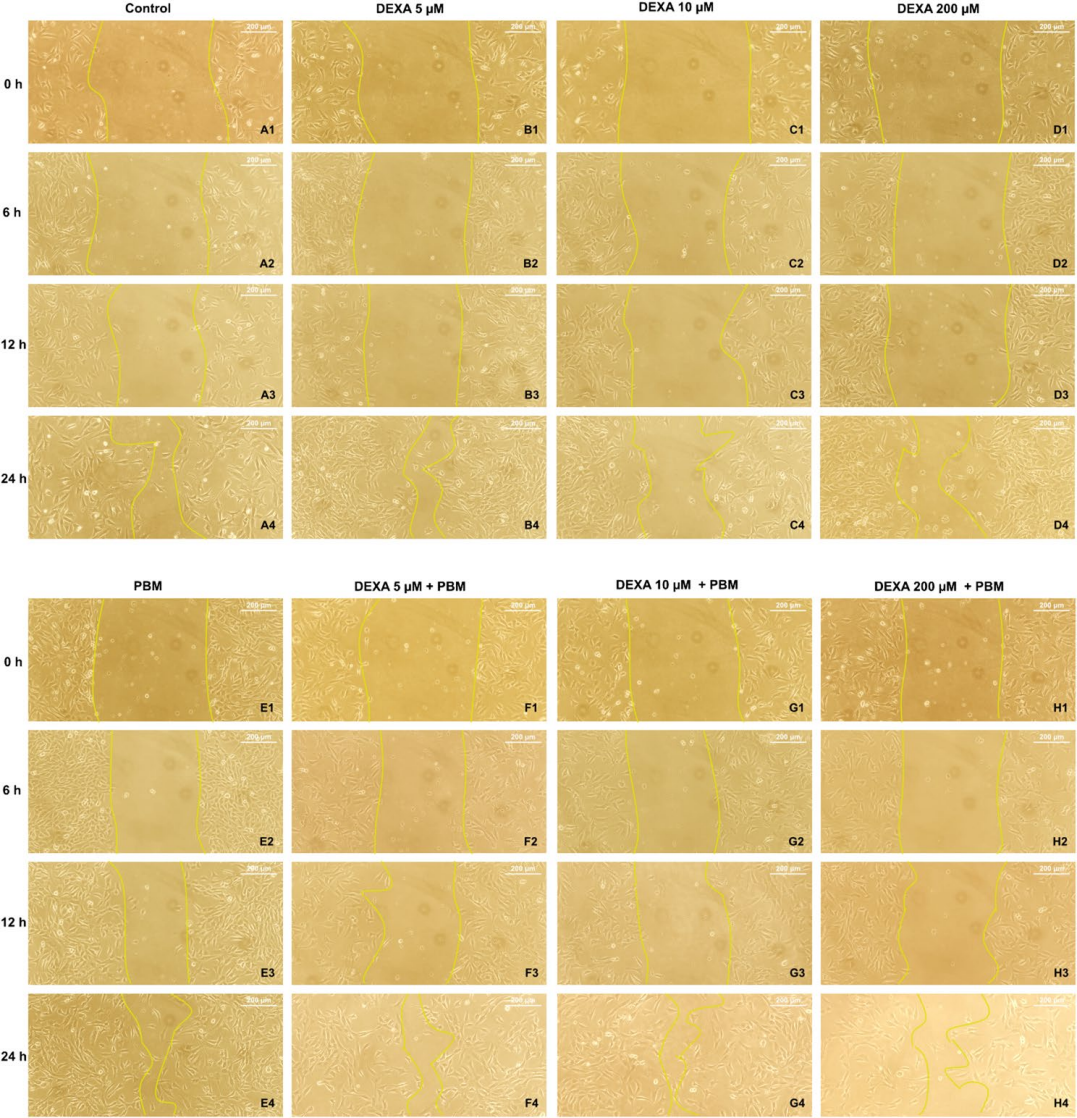
Representative images of the wound healing assay in C2C12 cells treated with DEXA and/or PBM. Treatments were applied at the beginning of the experiment, and wound closure was monitored at 0, 6, 12, and 24 h. Images were captured using an inverted microscope (Eclipse TE300, Nikon, Tokyo, Japan) at 10X magnification. Scale bar = 200 μm.

In the migration assay, wound closure progressed gradually across all groups, but with clear differences depending on the treatment (Figs. 5 and 6). After 6 h, the DEXA-treated groups (5, 10, and 200 µM) did not show significant differences in wound closure compared with the control group. In the groups treated with DEXA combined with PBM, a significant increase in wound closure was observed only for the DEXA 5 µM + PBM group when compared with the corresponding DEXA-only group.

After 12 h, DEXA reduced wound closure compared with the control group, with statistically significant reductions observed at 10 and 200 µM. Under these conditions, PBM partially attenuated this effect, and the DEXA 5 µM + PBM group showed significantly greater closure than the corresponding DEXA-only group.

At 24 h, all groups treated with DEXA, with or without PBM, exhibited reduced wound closure compared with the control group. However, within the same DEXA concentration, the DEXA + PBM groups generally showed higher closure values than their respective DEXA-only groups.

### IL-6 protein levels

**Fig. 7:**
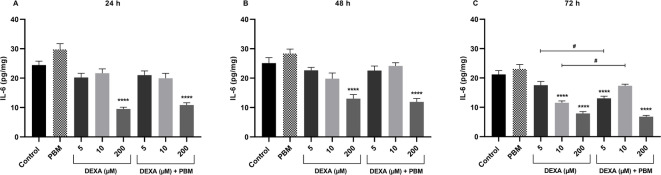
IL-6 levels in C2C12 cells across different experimental groups treated with DEXA, PBM, or their combination after 24 h (**A**), 48 h (**B**), and 72 h (**C**). Data are presented as mean ± SEM. Statistically significant differences compared to the control group are indicated by asterisks (*), whereas significant differences between groups treated with the same DEXA concentration are indicated by hashtags (#). Statistical analysis was performed using ANOVA followed by Tukey’s post hoc test: *p < 0.05; **p < 0.01; ***p < 0.001; ****p < 0.0001; #*p* < 0.05; ##*p* < 0.01; ###*p* < 0.001; ####*p* < 0.0001.

IL-6 levels showed a time- and dose-dependent reduction in response to DEXA (Fig. 7). At 24 h, all DEXA-treated groups exhibited lower values than the control, although a significant decrease was observed only at 200 µM, both alone and in combination with PBM. Groups treated with PBM alone and those exposed to 5 or 10 µM DEXA, with or without PBM, did not differ significantly from the control group. After 48 h, the same pattern remained, and only the DEXA 200 µM group, alone or in combination with PBM, showed a significant reduction in IL-6 levels. At the same time, the other concentrations did not differ from the control. After 72 h, significant reductions in IL-6 levels were observed in the DEXA 10 µM and 200 µM groups compared with the control. In the combined treatment groups, DEXA + PBM at 5 µM and 10 µM also showed significantly reduced IL-6 levels.

### TNF- α protein levels

**Fig. 8:**
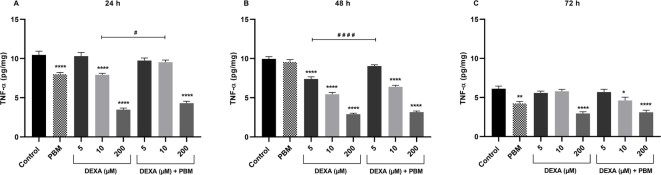
TNF-α levels in C2C12 cells across different experimental groups treated with DEXA, PBM, or their combination after 24 h (**A**), 48 h (**B**), and 72 h (**C**). Data are presented as mean ± SEM. Statistically significant differences compared to the control group are indicated by asterisks (*), whereas significant differences between groups treated with the same DEXA concentration are indicated by hashtags (#). Statistical analysis was performed using ANOVA followed by Tukey’s post hoc test: *p < 0.05; **p < 0.01; ***p < 0.001; ****p < 0.0001; #*p* < 0.05; ##*p* < 0.01; ###*p* < 0.001; ####*p* < 0.0001.

TNF-α protein levels are shown in Fig. 8. At 24 h, only the 10 and 200 µM DEXA groups showed a significant reduction in TNF-α levels, both alone and in combination with PBM, while 5 µM did not differ from the control. At 48 h, all DEXA-treated groups, with or without PBM, showed a significant decrease in TNF-α. At 72 h, the significant reduction in TNF-α persisted in the groups treated with 10 and 200 µM DEXA, both alone and in combination with PBM, whereas 5 µM did not differ from the control. PBM alone also showed a statistically significant reduction (Fig. 8).

## Discussion

Glucocorticoid-induced muscle atrophy remains a significant clinical concern due to its impact on muscle structure and function. In this study, PBM was able to lessen some of the cytotoxic effects of DEXA on C2C12 myoblasts, improving cell viability, proliferation, and early differentiation, as well as enhancing migration and influencing the production of inflammatory cytokines. These findings support the notion that PBM can provide protective and regenerative benefits even under glucocorticoid-induced stress. A wider range of dexamethasone concentrations (5-200 µM) was used to evaluate different levels of pharmacological stress in myoblasts. Concentrations of 5 and 10 µM were selected to represent moderate exposure levels, commonly associated with early alterations in cellular function, while the 200 µM concentration was included to simulate a more severe pharmacological stress condition^11,18,20^. This experimental approach allowed us to evaluate the potential of photobiomodulation to modulate cellular responses under different degrees of dexamethasone-induced impairment.

Viability analysis (MTT) showed that DEXA reduces C2C12 cell viability in a time- and dose-dependent manner, with minimal changes during the first 24 h and more pronounced decreases at 48 and 72 h, particularly at higher concentrations. A very similar trend has been reported in studies showing a progressive decline in cell viability as DEXA concentration increases and treatment duration is extended^17,18^. Similar to the compounds examined in these studies, such as S-Rg3 and the total protein extract of Panax ginseng, PBM in our study also showed a protective effect. When combined with DEXA, it consistently increased viability, although not enough to completely restore control levels, particularly at 200 µM, the concentration at which cell damage is most pronounced. This partial effect suggests that PBM helps mitigate the initial stress triggered by glucocorticoids. Another study reported a similar pattern, with increased viability following PBM, suggesting a direct stimulus on mitochondrial function^25^. The convergence of these findings reinforces the idea that, although it cannot fully reverse the effects of DEXA, PBM helps sustain cellular metabolism and lessens the magnitude of viability loss, particularly at intermediate drug concentrations.

Analysis of proliferation using the crystal violet assay showed that DEXA alone did not significantly affect C2C12 growth at any of the evaluated time points. In contrast, its combination with PBM consistently increased proliferation, with increases already evident at 24 h and more pronounced at 48 and 72 h. When comparing groups within the same concentration, the DEXA + PBM conditions, particularly at 5 and 10 µM, showed greater proliferation than the respective groups treated with DEXA alone. Taken together, these findings indicate that PBM exerts a stimulatory effect on cell proliferation, strengthening the proliferative response even in the presence of DEXA. Similar results have been reported in studies showing increased C2C12 proliferation following PBM (780 nm, 70 mW, 26,25 J/cm^2^, 1,05 J), particularly at 48 h, even when the cells were exposed to an unfavorable microenvironment induced by pro-inflammatory mediators released by M1 macrophages^25^. These findings suggest that, whether under pharmacological stress induced by DEXA or in inflammatory conditions driven by M1 macrophages, PBM retains its ability to promote myoblast proliferation, underscoring its pro-regenerative potential regardless of the type of cellular stress involved.

The apparent differences between proliferation and viability results can be explained by the distinct biological parameters assessed by the assays used. The MTT assay primarily reflects cellular metabolic activity rather than directly measuring cell number. The reduction of MTT to formazan occurs predominantly in mitochondria through the activity of mitochondrial oxidoreductases, making this assay highly sensitive to alterations in mitochondrial function and cellular bioenergetics. However, MTT reduction can also occur in other cellular compartments, including the endoplasmic reticulum, endosomal and lysosomal vesicles, the cytosol, and plasma membrane-associated redox systems via NADH- and NADPH-dependent oxidoreductive pathways, supporting its interpretation as an indirect indicator of cell viability rather than a strict measure of cell number^41,42^. In contrast, the crystal violet assay quantifies the total DNA content of adherent cells and therefore primarily reflects the number of cells present in the culture. Consequently, DEXA may impair mitochondrial metabolic activity without necessarily causing an immediate proportional reduction in cell proliferation. In addition, PBM has been reported to stimulate mitochondrial activity and increase ATP production, which may support cell cycle progression and contribute to the enhanced proliferative response observed in the PBM + DEXA groups. Thus, the combined interpretation of these assays suggests that PBM may primarily enhance cellular bioenergetics, which in turn supports proliferative activity even under DEXA-induced metabolic stress.

DEXA reduced the fusion index at all evaluated time points, whereas PBM alone enhanced myotube formation. When combined with DEXA, PBM did not fully restore control levels but partially mitigated the impairment, resulting in more elongated and better-organized cells during differentiation. This trend is consistent with previous studies in which PBM supported early stages of differentiation even within a pro-inflammatory environment^25^. Likewise, analyses using May–Grünwald and Giemsa staining have shown an increase in the number of myonuclei within the first 24 h, suggesting that PBM may stimulate early events preceding myoblast fusion^27^. Interventions capable of modulating stress-related pathways, such as treatment with acetyl-genistin, have also been shown to mitigate DEXA-induced myogenic impairment and preserve the fusion index^43^. Together, these findings suggest that although DEXA interferes with myogenic differentiation, modulatory strategies such as PBM may attenuate its effects and help sustain the progression of myogenesis.

An additional point to consider is that myogenic fusion in the present study was quantified using the fusion index, calculated as the proportion of nuclei incorporated into binucleated and multinucleated cells relative to the total number of nuclei, a method widely used to evaluate C2C12 differentiation^34,35^. Although this metric provides a reliable estimate of the extent of myoblast fusion, it does not fully capture structural aspects of myotube maturation. In the present study, qualitative morphological observations suggested partial preservation of myotube organization in PBM-treated groups; however, the average number of nuclei per myotube was not quantified. Future studies incorporating additional morphometric parameters, such as nuclei per myotube, may provide a more detailed characterization of myogenic maturation under DEXA exposure and PBM treatment.

Migration results showed that DEXA progressively impaired wound closure, with more pronounced effects at 12 and 24 h. PBM attenuated this impairment, increasing closure compared to groups treated with DEXA alone. However, irradiation did not completely restore motility to control levels, indicating that its effect remains partial in the presence of glucocorticoid-induced inhibition. PBM has also been linked to enhanced C2C12 migration, with LED at (850 nm, 40 or 70 mW, 0.13 J/cm2, 0.4 J) or laser (780 nm, 40 or 70 mW, 10 J/cm2, 0.4 J) irradiation, accelerating wound closure in the early hours and maintaining higher closure rates throughout the assay^27^. Similarly, PBM (660 nm, 14.08 mW, 0.04 cm^2^, 352 mW/cm^2^, 3.52 J/cm^2^, 0.1408 J and 780 nm, 17.6 mW, 0.04 cm^2^, 440 mW/ cm^2^, 4.4 J/cm^2^, 0.176 J) has been shown to promote wound closure in myoblasts exposed to Bothrops jararacussu venom, reinforcing its ability to stimulate migration even under damaging conditions^44^.

The partial recovery observed in the present study may be explained by the distinct cellular mechanisms underlying proliferation and migration. PBM enhances cellular bioenergetics through mitochondrial activation and increased ATP production, processes that are closely associated with cell cycle progression and proliferative responses^24,45^. In contrast, cell migration requires additional mechanisms such as cytoskeletal remodeling, regulation of focal adhesion complexes, and dynamic interactions with the extracellular matrix^46^. Glucocorticoids such as DEXA are known to interfere with several of these structural and signaling pathways, affecting cytoskeletal organization, protein turnover, and muscle cell homeostasis^22^. Consequently, although PBM may enhance cellular metabolism and support proliferative activity, the structural and signaling alterations induced by DEXA may limit the complete restoration of migratory capacity under the experimental conditions evaluated.

In the quantification of IL-6 and TNF-α, both cytokines showed a time- and dose-dependent decrease, particularly at the higher concentrations, reflecting the glucocorticoid’s suppressive effect on inflammatory pathways that are essential for the onset of myogenic differentiation. Although PBM did not fully restore control levels, it attenuated part of these reductions, most notably at 10 and 200 µM, preserving a minimal cytokine output that may help prevent an excessive blockade of the early stages of regeneration. This modulation is also consistent with findings showing that PBM (660 nm, 14.08 mW, 0.04 cm^2^, 352 mW/cm^2^, 3.52 J/cm^2^, 0.1408 J and 780 nm, 17.6 mW, 0.04 cm^2^, 440 mW/ cm^2^, 4.4 J/cm^2^, 0.176 J) reduced the excessive production of IL-6 and TNF-α in C2C12 myoblasts exposed to Bothrops jararacussu venom, contributing to the reestablishment of a cellular environment more conducive to repair^44^. Although the sources of damage differ between the models, both sets of results indicate that PBM acts by adjusting the inflammatory response, either by attenuating excessive activation or alleviating excessive suppression, suggesting an important regulatory role in preserving myogenic capacity under various stress conditions.

The findings of this study demonstrate that PBM mitigates the catabolic effects of DEXA in C2C12 myoblasts by preserving cell viability, supporting proliferation, maintaining early stages of differentiation, and modulating cytokines involved in myogenesis. Although it does not completely reverse the damage induced by the glucocorticoid, PBM reduces key features of DEXA-induced atrophy and emerges as a promising complementary strategy to help preserve skeletal muscle function and regenerative capacity.

Some limitations of the present study should be considered. Although the results demonstrate relevant cellular and inflammatory modulation in response to DEXA and PBM, the analyses performed do not allow identification of the intracellular signaling pathways involved in these responses. Myogenesis and muscle atrophy are regulated by complex molecular networks involving pathways associated with inflammation, protein synthesis, and proteolysis. Therefore, further studies incorporating molecular analyses of signaling pathways and regulatory proteins are necessary to better elucidate the mechanisms underlying the biological effects observed in this experimental model.

The relevance of these findings should also be considered in light of the high incidence of skeletal muscle injuries and the frequent clinical use of corticosteroids in their management. Although glucocorticoids are widely prescribed due to their potent anti-inflammatory effects, prolonged or repeated exposure may negatively affect skeletal muscle homeostasis, contributing to muscle atrophy and impaired regenerative capacity. In this context, the identification of therapeutic approaches capable of modulating these deleterious effects is particularly important. PBM, recognized as a safe, non-invasive and painless therapy that is easy to apply, represents an attractive therapeutic possibility. Further investigations are required to clarify the molecular mechanisms involved and to validate these findings in additional experimental models, which may contribute to the future development of safer strategies for the treatment of muscle injuries in patients who require corticosteroid therapy while minimizing the adverse effects of these medications.

## Electronic Supplementary Material

Below is the link to the electronic supplementary material.

Supplementary Material 1

## Data Availability

The authors declare that the data supporting the findings of this study are available within the paper and its Supplementary Information files. Should any raw data files be needed in another format, they are available from the corresponding author upon reasonable request.

## Declarations

### Use of AI tools

The authors used ChatGPT (OpenAI) to assist with English language editing and improvement of text clarity and readability. All scientific content, interpretation, and conclusions were developed and reviewed by the authors, who take full responsibility for the manuscript.
